# Multi-Modal Data Fusion for Quality Discrimination and Flavor Analysis of Commercial Oat Milk

**DOI:** 10.3390/foods15050936

**Published:** 2026-03-07

**Authors:** Leheng Jiang, Yuhao Cheng, Qiao Sun, Xiaoming Guo, Xiuping Dong, Yizhen Huang, Xiaojing Leng

**Affiliations:** 1 College of Food Science and Nutritional Engineering, China Agricultural University, Beijing 100083, China; 2 Shenzhou Space Biotechnology Group, Beijing 100086, China; 3 Shenzhen Key Laboratory of Food Nutrition and Health, Institute for Innovative Development of Food Industry, College of Chemistry and Environmental Engineering, Shenzhen University, Shenzhen 518060, China; 4 State Key Laboratory of Marine Food Processing and Safety Control, National Engineering Research Center of Seafood, School of Food Science and Technology, Dalian Polytechnic University, Dalian 116034, China

**Keywords:** oat milk, sensory attributes, physicochemical properties, product development

## Abstract

In this study, 10 popular commercial oat milk samples were analyzed for sensory quality and flavor chemistry using the Ideal Profile Method (IPM), electronic nose (E-nose), and gas chromatography-mass spectrometry (GC-MS). Based on consumer cognitive mapping of ideal products, samples were classified into “Ideal-like” and “Ideal-exceeding” categories. Ideal-like products exhibited light white appearance, pronounced oatiness, moderate sweetness and burntness, and low graininess, presenting a balanced flavor profile, whereas Ideal-exceeding samples surpassed consumer expectations in sweetness or graininess intensity, delivering stronger sensory stimulation. Furthermore, sensory differentiation among categories primarily stemmed from synergistic effects of lipid oxidation levels (e.g., 3,5-octadien-2-one) and physical stability (fiber and protein content affecting particle size distribution). This classification framework reveals that ideal sensory quality can be achieved through diverse physicochemical pathways in commercial oat milk, providing theoretical guidance for product formulation optimization and quality standardization.

## 1. Introduction

In recent years, consumer concerns regarding milk allergy, lactose intolerance, weight management, and hypercholesterolaemia—together with the growing acceptance of vegan dietary practices—have collectively accelerated the development of plant-based dairy alternatives. Plant-based milk beverages (PBMs) are now widely regarded as a principal substitute for bovine milk because they are free of dairy allergens, lactose, and cholesterol, and are rich in bioactive phytochemicals [1,2,3]. Oat milk, in particular, has become the second-largest PBMs category, recording a compound annual growth rate (CAGR) of 131.9%; its market share is projected to continue expanding through 2027 [4]. This ascendancy is underpinned by two key factors. First, oat β-glucan exhibits well-documented hypocholesterolaemic and glycemic-regulating properties. Second, oat milk possesses a neutral sensory profile that lacks the “beany” off-notes commonly associated with soymilk, thereby aligning more closely with consumer taste preferences. As a globally cultivated crop with >2000 years of agricultural history, oat ranked sixth among cereals in 2022, with world production reaching 24.998 million metric tons. This reliable and abundant raw-material base provides a secure supply chain for the sustained growth of the oat-milk industry [5,6].

Despite a buoyant market outlook, commercial oat beverages continue to encounter persistent quality deficits and consumer-acceptance barriers [7]. Specifically, these challenges are primarily driven by inconsistencies in sensory attributes, such as flavor, texture, and physicochemical stability. Common defects—including batch-to-batch flavor drift, residual bitterness, thin viscosity, and a powdery coating—immediately degrade the drinking experience [8]. Volatile organic compounds are the primary culprits: inherent grassy, beany, astringent, and bitter notes originate mainly from lipid-oxidation aldehydes, hexanal foremost among them [9], whereas heat-induced Maillard reactions generate pyrazines and furans that further complicate the aroma profile. These off-flavors, in turn, hinder consumer acceptance, especially when they exceed perceptible sensory thresholds [10]. Similarly, texture-related issues, including low protein content and weak gel networks, prevent the development of a spoonable consistency typically associated with dairy milk [11]. This results in a watery texture, low storage moduli, and frequent whey syneresis, which diminish the mouthfeel and overall consumer experience [12]. Negative early-generation memories further bias perception via cognitive assimilation, suppressing acceptance of improved successors [13].

Moreover, individual scoring differences in consumer sensory evaluations are becoming increasingly prominent. Different consumer groups often hold divergent internal scoring standards and sensory expectations for various products based on their performance in flavor complexity, texture fullness, and physicochemical stability. While many studies have explored the general acceptance of PBMs [14,15], few have rigorously examined the sensory gaps between currently available products and their ideal versions from a consumer perspective. Thus, it remains a problem to explicitly define the sensory direction of improvement from the consumer perspective. The Ideal Profile Method (IPM) is a method which aims at acquiring sensory data from consumers [16]. This method, effectively applied in small sample studies, enables the identification of sensory attributes that are most important to consumers, allowing producers to better target and refine those aspects [17]. Additionally, through the use of sensory evaluation scales, IPM allows for the expression of subtle differences in product evaluations [18]. However, the consistency of ideal data must be considered [19], as consumer preferences may vary by demographic. To accurately identify these diverse ideal reference points and determine the intrinsic ideal standards of consumers, it is essential to introduce IPM, which helps establish individualized “ideal product” profiles and systematically deconstruct the heterogeneity of sensory preferences, providing methodological support for precise product optimization.

In this study, we specifically focus on the preferences of the youth demographic, a group that typically exhibits a greater openness to new products and is more sensitive to sensory innovations. By combining IPM with physicochemical analysis tools such as the electronic nose (E-nose) and gas chromatography-mass spectrometry (GC-MS), this study aims to offer actionable insights into the sensory qualities of commercial oat milk. This approach not only reflects consumer demands but also guides the development of a more consumer-centric plant-based milk alternative.

## 2. Materials and Methods

### 2.1. Sample Collection

Ten commercial oat milk products were purchased from the official JD.com (Beijing, China) e-commerce platform between March and May 2025, based on their top 10 sales ranking on the platform. All samples were unopened products with at least 80% of their shelf life remaining. Upon delivery, samples were immediately transported to the laboratory and stored in their original sealed packaging at 4 °C in darkness until analysis. Samples were analyzed within 7 days of receipt.

Samples were coded (M1–M10) for systematic identification during instrumental analysis. The correspondence between sample codes and product names is provided in Table 1. All measurements were performed in triplicate using freshly opened cartons for each replicate to eliminate variability from repeated sampling.

### 2.2. Sensory Evaluation

As described by McCarron et al. [20], descriptive sensory profiling was carried out over the course of 2 weeks, using the trained sensory panel at China Agricultural University. Vocabulary development and training sessions were conducted in a discussion room, where panelists were familiarized with descriptors for sensory attributes such as appearance, aroma, taste, texture, and overall liking. The vocabulary was developed through group discussions, ensuring consistency in its application. Finally, 12 sensory attributes were evaluated (Table S1).

A total of 40 untrained participants were involved in the sensory assessment; the sensory attributes were presented in a Rate-All-That-Applied (RATA) with a 15-point scale, where 0 = ‘not present’, and 15 = ‘extremely present’. After rating the commercial products, the questions were repeated, but the panelists were told to focus on their ideal version of oat milk [17,21]. Sensory attributes were also evaluated using a 9-point just-about-right (JAR) scale, with anchor points “much too little” and “much too pronounced” [22].

### 2.3. Physicochemical Characterization of Oat Milk Samples

#### 2.3.1. Color and Soluble Solids

Color analysis of oat milk samples was carried out using a 3nh Chroma Meter NR-200 (Shenzhen 3nh Technology Co., Ltd., Shenzhen, China), with slightly modifications as described by Paliwal et al. [23]. The recorded parameters were lightness (L*), red–green (a*), and blue–yellow (b*) intensities. 150 mL of the sample was placed into a square plate, and the color was measured three times from each duplicate sample. The samples were placed in a black box to avoid interference, and the instrument was calibrated prior to testing. The total soluble solid of the samples was determined by a hand refractometer (PAL-1, Atago, Tokyo, Japan, reading at 20 °C) and were expressed as °Brix [24].

#### 2.3.2. Total Titratable Acidity and pH

Total titratable acidity (TTA) was determined by titrating 20 mL of oat milk with 0.1 mol/L NaOH (Shanghai Macklin Biochemical Co., Ltd., Shanghai, China) to pH 6.5 [25]. Results were expressed as g lactic acid equivalents per 100 mL of sample, calculated based on the volume of NaOH consumed. pH was measured directly using a calibrated digital pH meter (PHS-3C, Leici, Shanghai, China) [26].

#### 2.3.3. Particle Size

The particle size of the sample was measured by a laser diffraction particle analyzer (BT-9300ST, Dandong Bettersize Instruments Ltd., Dandong, China) with the method reported by Hu et al. [27]. The continuous phase was oat milk, and the dispersed phase was water (refractive index 1.33).

#### 2.3.4. Rheological Tests

A rheometer (MCR 302, Anton Paar GmbH, Graz, Austria) was used to analyze the rheological properties of oat milk [28]. Measurements were conducted at a constant temperature of 37 °C to simulate the temperature in the human mouth, using a parallel plate geometry with a diameter of 40 mm. The sample was carefully loaded onto the rheometer plate, and the gap between the plates was adjusted to 1.0 mm. Prior to measurement, all samples were equilibrated at the test temperature for 2 min to ensure thermal stability. Steady shear tests were carried out over a shear rate range of 0–100 s^−1^, and the corresponding shear stress and apparent viscosity were recorded. The flow behavior of the samples was characterized by fitting the data to the power-law model (Equation (1)):

(1) $$\tau =K\cdot {\dot{\gamma }}^{n}$$

where *τ* is the shear stress (Pa), *K* is the consistency coefficient (Pa·s^n^), *n* is the flow behavior index (dimensionless), and $\dot{\gamma }$ is the shear rate (s^−1^).

#### 2.3.5. Physical Stability

The physical stability of the oat-based beverage was evaluated using an analytical centrifuge (LUMiSizer^®^, LUM GmbH, Berlin, Germany), as described by Mäkinen et al. [29]. During the analysis, samples were exposed to centrifugal forces while the entire sample cell was illuminated with near-infrared light. The transmitted light intensity was continuously recorded as a function of time and position along the sample length, allowing the clarification and sedimentation processes to be monitored in real time. The separation rate was calculated as the slope of the integrated transmission versus time curve. Measurements were conducted at 24 °C under sequential centrifugal conditions of 140 rcf for 30 min, followed by 1260 rcf for 60 min.

#### 2.3.6. E-Nose Analysis

A PEN3 portable electronic nose analyzer (Airsense Analytics GmbH, Schwerin, Germany) was used [21], which consists of an array of 10 metal oxide gas sensors (Table S2). 5 g of each deodorized sample and the control group were weighed and placed in a 20 mL headspace vial, tightly sealed with a cap and covered with a sealing film for detection using the portable electronic nose. Each group of samples was tested in triplicate, with a blank experiment conducted between every two groups of samples.

#### 2.3.7. Volatile Analysis

VOCs in oat milk were extracted via headspace solid-phase microextraction (HS-SPME) and analyzed as described previously [30,31]. We used 5 g of oat milk with 1 μL of internal standard solution (1 mg/mL naphthalene-d_8,_ Shanghai Macklin Biochemical Co., Ltd., Shanghai, China) in a 20 mL headspace vial and performed SPME using a DVB/CAR/PDMS fiber (Supelco, Bellefonte, PA, USA). We equilibrated the sample at 50 °C for 5 min in a water bath, then extracted for 45 min.

The analysis was performed using an 8890-5977B GC-MS system (Agilent Technologies, Santa Clara, CA, USA) with HP-5MS UI column (30 m × 0.25 mm × 0.25 μm, Agilent Technologies, Santa Clara, CA, USA). The SPME was desorbed at 250 °C for 5 min, and the initial heating temperature was held at 45 °C for 3 min. The GC column was heated from 45 °C to 250 °C at a rate of 5 °C/min and held at 250 °C for 10 min.

### 2.4. Statistical Analyses

The quantitative data for each compound identified in the physicochemical measurements were analyzed by one-way analysis of variance (ANOVA) using XLSTAT Sensory (Version 2022.5.1.1388, Addinsoft, Paris, France). For those compounds or physical parameters exhibiting a significant difference in the ANOVA, Tukey’s honest significant difference (HSD) test was applied for multiple pairwise comparisons [32].

## 3. Results

### 3.1. Sensory Analysis

**Figure 1:**
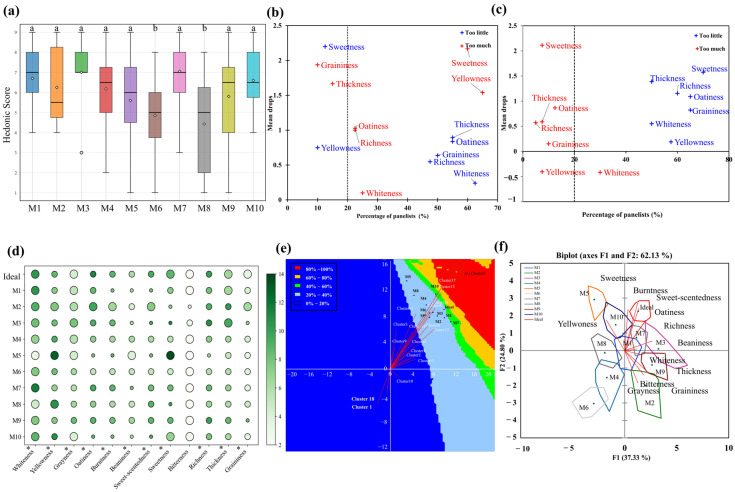
Sensory evaluation of oat milk samples. Note: (**a**) Hedonic biplot; (**b**) Total mean drops of M6; (**c**) Total mean drops of M8; (**d**) Bubble plot of sensory attributes; (**e**) Preference map of samples and (**f**) PCA biplot with bootstrap hulls. The size of the bubble represents consistency among panelists in (**d**). Commercial oat milk products: M1, Daily Box Organic; M2, Daily Box High-Fiber Original; M3, Daily Box Golden; M4, OATLY Standard; M5, OATLY Original; M6, OATLY Barista Edition; M7, Feichangmai Organic; M8, Songyuru Low-Fat High-Calcium; M9, Fixbody; M10, MILK LAB. * indicates a significance level alpha = 0.05. Different lowercase letters indicate statistically significant differences between groups (*p* < 0.05, Tukey’s HSD test); In (**b**,**c**), red “+” denotes “too much” and bule “+” denotes “too little” attribute intensity relative to the optimal level.

The sensory characteristics of oat milk are crucial factors influencing consumer preference. The sensory evaluation of samples revealed that M6 and M8 had the lowest overall liking (Figure 1a). Just-about-right (JAR) scales were therefore applied to identify the sensory attributes most strongly associated with product acceptance [33]. The penalty analysis (Figure 1b,c) revealed that the reduced acceptability of M6 was mainly attributed to excessive sweetness combined with insufficient oatiness and thickness, whereas the low preference for M8 was primarily driven by weak oat flavor, pronounced graininess, and inadequate thickness. These results indicate that imbalances in sweetness, viscosity, and oat-related flavor are key factors underlying reduced consumer acceptance.

Panelists further provided detailed descriptions of the flavor and texture differences using the RATA scales. Significant differences were observed for all attributes except bitterness. According to Waehrens et al. [17], when the mean intensity score of a given attribute in the ideal product exceeds that of commercial samples, consumers desire a stronger expression of this attribute, whereas the opposite indicates a preference for a reduced intensity. As illustrated in Figure 1d, the ideal version was characterized by a moderately white appearance, strong oatiness, moderate sweetness, and low graininess. Note that the gaps between commercial versions and the ideal version were >1.0 units on the RATA intensity scale, suggesting the oatiness of commercial versions must be improved. Among the samples, M1, M7, and M10 exhibited intensity patterns for sweetness, oatiness, and thickness that were closest to the ideal profile, whereas M6 showed markedly lower sweetness and M8 deviated substantially in appearance and texture.

To gain further insights into consumer preferences, consumer cluster analysis was conducted (Figure 1e). The black dots represented the results of principal-component analysis (62.13%). Distribution of samples and clusters indicated that the ideal version was given overall preference ratings between 20% and 40% of the average, and was preferred by cluster 6 and 16. Based on their proximity to the ideal point in the sensory preference map, M1, M3, M7, and M10 were identified as the closest approximations to the ideal oat milk, whereas M2, M4, M5, M6, M8, and M9 were positioned at greater distances. This were aligned with the PCA biplot with bootstrap hulls (Figure 1f). The bootstrap hulls non-overlapping indicated robust differentiation between sensory attributes. Overlapping regions for M1–M7 and M1–M10 suggested high sensory similarity, with oatiness, sweetness, and richness vectors oriented toward both these samples and the ideal product. In contrast, the grainiess vector pointed toward M2 and M9, explaining their greater deviation from the ideal profile.

Across the ten selected oat milk samples, clear sensory patterns emerged. As shown in Table 1, M1, M3, M7 and M10—typical representatives of organic oat milk—presented a markedly whiter appearance, pronounced oatiness, moderate sweetness and burnt notes, together with low graininess, matching consumer expectations for the ideal version. These samples were categorized as ideal-like products, as they closely matched the sensory attributes consumers expect in an ideal version of oat milk. On the other hand, other samples, such as M2, M5 and M9, were classified as ideal-exceeding, as they not only met the ideal sensory criteria but also exceeded expectations in certain attributes (e.g., sweetness). Overall, consumers preferred oat milk with light white appearance, moderate sweetness and graininess, enhanced oat-like and roasted flavors, consistent with previous finding by Waehrens et al. [17]. This preference reflects consumer awareness of health trends, aligning with the motivations to consume PBMs, even though humans naturally tend to favor sweetness [34].

### 3.2. Instrumental Analysis

To further define the characteristics of the ideal version we also investigated the physicochemical properties of these 10 samples. This analysis provides a more comprehensive understanding of the factors that influence consumer preferences and helps to refine the ideal version profile.

#### 3.2.1. Basic Physicochemical Properties

The nutritional composition of oat milk is one of the key reasons for its popularity. However, the various oat milk samples exhibited considerable differences in their characteristics. As shown in Table 2, the Brix values of samples ranged from 9.6% to 14.77%. Among the samples, M9 exhibited the highest soluble solids content (14.77 ± 0.77%), likely due to its higher carbohydrate content and enzymatic processes [24]. During enzymatic processes, the degradation of the biomacromolecule is facilitated, promoting the hydrolysis and solubility of starch components, finally enhanced the Brix as well as sweetness [27,35]. Notably, ideal-like products showed relatively moderate Brix and pH values. Combined with the results of sensory analysis, oat milk with lower Brix may have less graininess and moderate sweetness.

In terms of color, all samples exhibited a classic slightly brownish tinge although considerable variation was observed [36]. The lightness (L*) of oat milk ranged from 59.84 ± 0.31 to 69.36 ± 0.31, which wass lower than the typical L* value of 75–80 for cow’s milk [37]. Ideal-like products (M1, M3, M7, and M10) exhibited higher L* values, indicating lighter colors, while other samples (M5, M9), with roasted aroma labels, showed higher b* values, reflecting more intense yellow hues. This can be attributed to melanoidins formed during the Maillard reaction, a process occurring during thermal processing that imparts darker and more yellowish tones [38]. However, the characteristic color of oat is beige, which aligns with the strong white and moderate yellow appearance of the ideal version described in Section 3.1. These color differences also highlight the distinction between ideal-exceeding preferences and the remaining samples.

#### 3.2.2. Physical Properties

**Figure 2:**
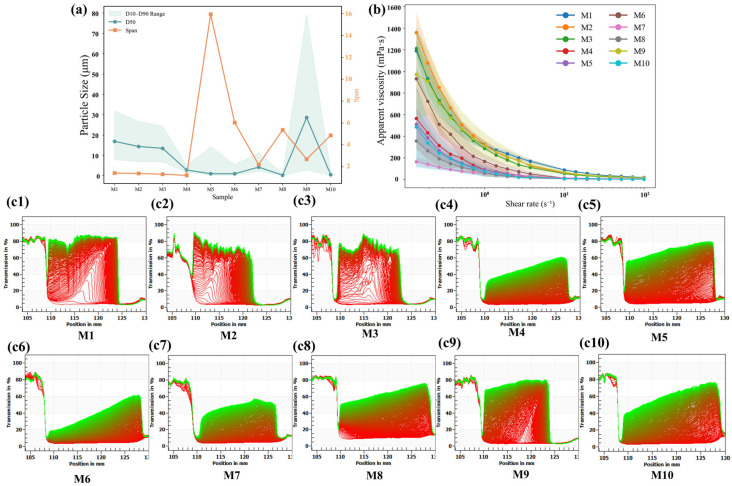
Comprehensive physical characterization of oat milk samples. Note: (**a**) Particle size distribution. (**b**) Rheological properties; different colored shadows represent standard deviation bands for different samples. ((**c1**)–(**c10**)) Physical stability of different commercial brands (M1–M10). Red lines represent the projection profile at the first measurement, while green lines indicate the final measurement after. Subfigures ((**c1**)–(**c10**)) show physical stability of commercial oat milk brands M1–M10, respectively. M1, Daily Box Organic; M2, Daily Box High-Fiber Original; M3, Daily Box Golden; M4, OATLY Standard; M5, OATLY Original; M6, OATLY Barista Edition; M7, Feichangmai Organic; M8, Songyuru Low-Fat High-Calcium; M9, Fixbody; M10, MILK LAB.

Particle size is crucial for maintaining the stability of oat milk [39]. As shown in Table 3,ideal-like products (M3, M7, M10) exhibited moderate *D_[4,3]_* values, with M9 showing an especially high *D_[4,3]_* value (Figure 2a). M5 had the highest span, the broadest particle size distribution, and poorer uniformity.

The rheological properties of a beverage are important parameters, with appropriate viscosity provideng a rich and full mouthfeel. The relationship between shear rate and apparent viscosity is shown in Figure 2b, with fitting model parameters presented in Table 3. The apparent viscosity of these fluids decreased with increasing shear rate and had a flow behavior index (*n*) less than 1; all samples exhibited shear-thinning behavior, classifying them as pseudoplastic non-Newtonian fluids [40]. When consumed or swallowed, the shear rate increases, and viscosity rapidly decreases, giving the product a smooth texture and ease of swallowing [29]. Among the ideal-like samples, M1 and M3 showed high apparent viscosity, significantly higher than reported values [41], while M10 exhibited lower apparent viscosities.

The optical centrifugation system is another real-time visual monitoring method for phase separation in oat milk. By measuring the light transmittance during centrifugation, the stability coefficients for 10 oat-milk samples were obtained (Figure 2(c1)–(c10)). The red line in the figure represents the projection profile at the first measurement. A denser red-line profile corresponds to a lower phase-separation rate, thereby indicating superior stability. As illustrated in Figure 2c, the transmittance of all samples showed a progressive increase with extended centrifugation time. And the transmittance rate at the bottom of each tube was lower than the rest, indicting phase separation induced by sample instability during centrifugation. The projection profiles of most commercial oat milks are trapezoidal in shape [42], indicating good stability. While most samples showed similar shape, M6 exhibited the best centrifugal stability. In contrast, the sparse and irregular red-line patterns observed for M1 and M3 indicated poor physical stability. This behavior suggests that higher levels of fiber and protein, as observed in samples M1–M3 (Table 1), may promote particle aggregation, resulting in larger particle sizes and reduced stability [27,43]. By contrast, M10, characterized by relatively lower fiber and protein levels (<0.6 g/100 g), exhibited smaller particle sizes, which likely contributed to its more stable appearance and mouthfeel during sensory evaluation.

Although higher apparent viscosity can theoretically retard particle sedimentation according to Stokes’ law and thereby enhance physical stability [44], the results of this study indicate that viscosity alone is not sufficient to ensure stability in oat milk systems. Despite their relatively high viscosities, samples M1–M3 still exhibited poor stability, most likely due to aggregation induced by elevated fiber and protein contents [35,40,45]. In contrast, samples M4 and M6, which combined fiber with higher fat levels, showed superior centrifugal stability, supporting previous findings that fat can mitigate particle aggregation and reduce phase separation in plant-based beverages [46].

Notably, even within the ideal-like group, significant differences in physical stability were observed. While M1 and M3 exhibited pronounced phase separation and poor centrifugal stability, M7 and M10 demonstrated comparatively more stable behavior. This indicates that achieving a sensory profile close to the ideal does not necessarily rely on a single physicochemical mechanism. These results suggest that, among the commercial samples evaluated in this study, multiple physicochemical pathways may lead to an ideal-like sensory perception, but only some of them are compatible with long-term physical stability.

#### 3.2.3. Volatile Analyses

**Figure 3:**
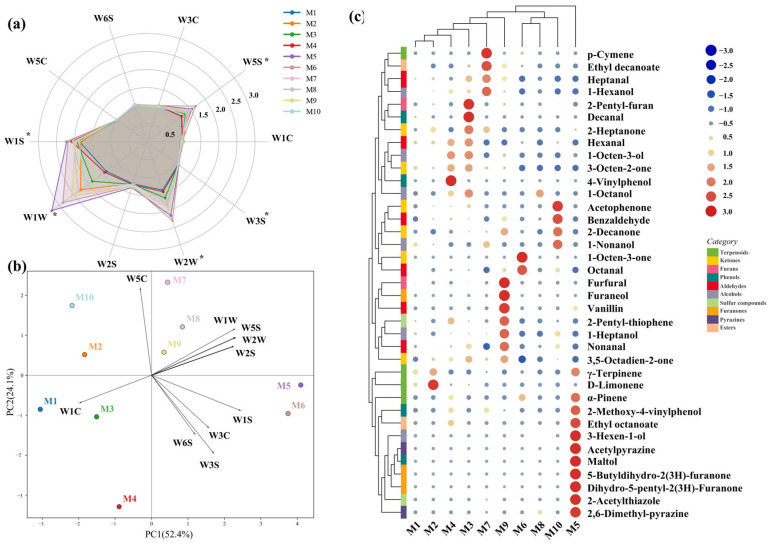
Aroma profile analysis of oat milk samples. Note: (**a**) E-nose radar, (**b**) E-nose PCA, (**c**) GC-MS heatmap. M1, Daily Box Organic; M2, Daily Box High-Fiber Original; M3, Daily Box Golden; M4, OATLY Standard; M5, OATLY Original; M6, OATLY Barista Edition; M7, Feichangmai Organic; M8, Songyuru Low-Fat High-Calcium; M9, Fixbody; M10, MILK LAB. * indicates significant differences among samples (*p* < 0.05).

Aroma is an important factor influencing the sensory quality of oat milk and serves as a key indicator of product acceptability. The e-nose provided an overall profile of volatile organic compounds (VOCs) and reflect the differences between different samples [47]. As shown in the radar chart (Figure 3a), M5 and M6 generated the strongest signals on most channels, followed by M9. Additionally, the PCA plot (Figure 3b), with the first two principal components explaining more than 70% of the variance, effectively illustrated the differences in flavor characteristics among the oat-milk samples. The first principal component (PC1) was dominated by sensor W5C, selective for aromatic compounds and alkanes, with M5 and M6 exhibiting a high positive score, whereas ideal-like products showed markedly high negative scores on PC1.

GC-MS further revealed flavor differences among the various oat milk brands (Table 4). A total of 37 volatile compounds were identified in the 10 oat milk samples, including aldehydes (6), alcohols (6), ketones (6), phenols (5), terpenoids (4), furanones (3), pyrazines (2), furans (2), sulfur compounds (2), and esters (1). Among these, aldehydes, alcohols, and ketones were the most abundant, while terpenoids and esters showed the least diversity, indicating that aldehydes, alcohols, and ketones are the major contributors to the aroma of oat milk. Table 4 presents the identification methods and odor descriptions of these compounds, and Figure 3c further illustrates the similarities and differences in compound profiles across the samples through a clustering heatmap. Further details are provided in Supplementary Table S3.

Hierarchical clustering analysis based on GC–MS data revealed distinct differences in volatile profiles among the oat milk samples (Figure 3c). Both the samples and volatile compounds were clearly separated into several clusters, indicating pronounced heterogeneity in aroma composition. Notably, several samples classified as ideal-like based on sensory evaluation (M1, M3, and M7) were grouped within the same cluster, suggesting that this cluster may represent a shared volatile profile associated with ideal-like oat milk. Within this cluster, compounds such as aldehydes, alcohols, furans, and terpenoids [8,48] exhibited broadly comparable relative intensities, supporting their potential role in anchoring ideal-like sensory expectations. Aldehydes and alcohols, known for their almond and nutty notes, are key aroma compounds in many cereals. Terpenoids are key distinguishing features of oat milk [8,49,50]. As the natural organic compounds sensitive to heat processing, they are often present in fresh oats [51]. The presence of high levels of terpenoids in this cluster contributed woody aromas, consistent with the typical oat aroma. [51,52]. These shared volatile features may contribute to the perception of typical oat aroma and flavor balance that aligns with consumer preferences identified in the ideal evaluation.

In addition, M10 was separated from the main ideal-like cluster but still met the criteria for ideal-like classification based on sensory cognition. This separation implies that ideal-like sensory perception may be achieved through alternative volatile compositions, highlighting the existence of multiple aroma pathways leading to similar consumer acceptance within the studied samples.

The development of the aroma, specifically for oat products, requires an intervention of heat treatment [38]. Pyrazines and furanones are key sources of nutty and roasted aromas. The presence of them beyond the ideal-like cluster may be due to heat treatment during oat processing [51,53], contributing strong flavor to the M5. Aside from artificially added flavor enhancers and thermal processing, most key VOCs in oat milk originated from lipid oxidation [52,54]. Both enzymatic and non-enzymatic pathways significantly influence the flavor profile of oat milk by generating a range of volatile compounds. Volatile aldehydes such as hexanal, are further broken down by hydroperoxide lyase from 9- and 13-hydroperoxide linoleic acid [55]. These aldehydes play a significant role in shaping the fresh and grassy flavor of oat milk, as observed in ideal-like products. Additionally, non-enzymatic oxidation, triggered by free radicals, metal ions, or light, also contributes to key flavor compounds. For example, 2-pentyl furan, a major product of this pathway, imparts a beany, earthy note to oat milk [44,56]. Thus, the concentrations of hexanal and 2-pentyl-furan in samples reflected the degree of enzymes in different oat milk and preferred by customers (Section 3.1), indicating ingredient freshness..

Both the e-nose and GC-MS indicated the clusters of different types of oat milk. They captured shared aroma characteristics associated with ideal-like oat milk perception, while also revealing internal variability among samples. Ideal-like products (M1, M3 and M7) were characterized by a delicate, fresh, almond-like aroma, primarily contributed by aldehydes, alcohols, furans, and terpenoids.

### 3.3. Integrated Interpretation of Sensory–Chemical Relationships

**Figure 4:**
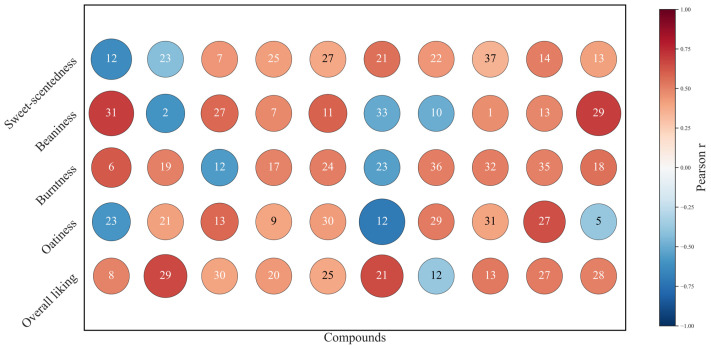
Pearson correlation bubble map between key volatile compounds and target sensory attributes. Note: A composite matrix was constructed that included the target attributes together with the top ten key VOCs (compound numbers as in Table 4). Bubble size and color scale indicated the magnitude and sign of the Pearson correlation coefficient, respectively; red denotes positive and blue denotes negative correlations.

Several studies have demonstrated that sensory properties of PMBs play a significant role in consumer influencing choice [57,58]. To further explore how volatile profiles relate to sensory perceptions in consumer-defined ideal oat milk, which is characterized by enhanced oatiness, reduced graininess, and moderated sweetness, a Pearson correlation analysis was applied (Figure 4). Based on consumer preference scores and fused GC–MS data, a composite matrix was constructed, incorporating target sensory attributes alongside the top ten representative volatile organic compounds (VOCs).

Oatiness, a key sensory attribute associated with the ideal version of oat milk, plays a crucial role in consumer acceptance. It was typically characterized by green, fatty and mushroomy aromas that consumers often associate with the “natural” flavor of oats [56]. In this study, oatiness was positively correlated with specific VOCs, particularly 3,5-octadien-2-one (compound **13**), 1-octanol (compound **27**), and 1-heptanol (compound **29**). These compounds, derived from lipid oxidation and fermentation processes, are well known for their contribution to the characteristic oat flavor, providing a creamy, cereal-like aroma that enhances the perception of oat milk.The importance of these VOCs in shaping the ideal sensory profile of oat milk aligned with previous studies that describe oat-like notes as being dominated by aldehydes and alcohols [59]. The positive correlations between these VOCs and oatiness suggest that the ideal version of oat milk is not only defined by its inherent oat flavor, but also by the presence of specific volatile compounds that contribute to its sensory complexity. Interestingly, the VOCs associated with oatiness overlap with those that contribute to sweetness and burntness, indicating that these attributes overlap with those contributing to sweetness and burntness, indicating that these attributes do not exist in isolation but interact to create a balanced flavor profile.

While oatiness remains a dominant characteristic of the ideal oat milk, the role of burntness in shaping consumer preferences was also observed. Moderate to high levels of burntness, typically associated with roasted and caramelized aromas, were found to reach consumer desires. VOCs such as furanones (compounds **35**, **36**), 2-acetylthiazole (compound **18**), and 4-vinylguaiacol (compound **6**), which are products of Maillard reactions during thermal processing [50], were positively correlated with burntness and played a complementary role in enhancing the overall flavor profile [59]. Notably, 4-vinylguaiacol (compound **6**), a key Maillard product, exhibited the strongest correlation with burntness, highlighting the importance of roasting and thermal processes in shaping the sensory appeal of oat milk.

An intriguing finding in this study was the dose-dependent nature of these volatile compounds. While compounds like 1-heptanol and 1-octanol are positively associated with oatiness, they are also involved in the formation of less desirable flavors such as beaniness [60]. This supports previous suggestions that aldehydes and alcohols can contribute to both desirable and undesirable flavor profiles, depending on their concentration [61]. Specifically, γ-terpinene (compound **2**), typically associated with fresh, citrus-like notes, was negatively correlated with beaniness, indicating that its presence may help mitigate undesirable off-flavors commonly associated with oat milk. This inverse relationship highlights the role of γ-terpinene in balancing the overall aroma profile, where its fresh, clean characteristics counteract the more unpleasant, beany notes that are generally regarded as off-putting by consumers [31,62].

Moreover, the attribute of sweetness was positively correlated with 1-nonanol (compound **21**), which is known to impart fruity and sweet aromas, contributing significantly to the perceived sweetness of oat milk [38]. Interestingly, 1-octen-3-one (compound **12**), which has an earthy and musty aroma, was negatively correlated with sweetness, indicating that its presence detracts from the sweetness of the product. This finding further supports the notion that while certain aldehydes contribute to desirable sweet notes, others may introduce undesirable flavors depending on their concentration.

Regarding overall liking, alcohols and ketones, including 1-heptanol (compound **29**), 1-nonanol (compound **21**) and 3,5-octadien-2-one, were key VOCs positively correlated with consumer acceptance. This compounds are associated with lipid oxidation [52,54], which underscore its critical role in flavor development. The positive association between 3,5-octadien-2-one and overall liking aligned with its strong correlation with oatiness, an attribute previously identified as a key driver of ideal-like perception in this study.

Overall, the correlation analysis suggested that only a subset of volatile compounds is closely associated with sensory attributes defining consumer-perceived ideal oat milk. These associations complement the GC–MS clustering results, highlighting perceptually relevant aroma signals shared among ideal-like products, while also indicating that differences in sensory acceptance cannot be attributed to volatile composition alone. Among the ten commercial samples analyzed, compounds such as 3,5-octadien-2-one, 1-octanol derived from lipid oxidation and 4-vinylguaiacol from Maillard reactions jointly shape the ideal version profile.

## 4. Conclusions

This study systematically evaluated the sensory attributes, physicochemical properties, and volatile profiles of ten commercial oat milk samples using an ideal-based framework. Based on ideal ratings, samples were separated into two clusters: ideal-like and ideal-exceeding. Sensory evaluation revealed that samples with light whiter apperance, moderate sweetness and graininess, more pronounced oat-like and burnt flavors were preferred by consumers.

Although ideal-like products exhibited noticeable differences in physical properties, their sensory profiles were highly consistent, indicating that multiple physicochemical pathways can converge toward an ideal sensory perception. E-nose and GC–MS identified aldehydes, alcohols, furans, and terpenoids as key contributors to ideal aroma. Pearson correlation analysis revealed that volatile compounds from lipid oxidation and thermal processing were associated with consumer-preferred attributes, including oatiness, sweetness, and burntness. Lipid oxidation products, such as 3,5-octadien-2-one, were strongly correlated with oat-like perception. Overall, this study provides new insights into the sensory–chemical mechanisms underlying consumer acceptance of oat milk. Future studies incorporating gas chromatography–mass spectrometry–olfactometry (GC–MS–O) and expanded sample sets are warranted to further elucidate the perceptual relevance of individual aroma-active compounds and to improve the generalizability of these findings.

## Figures and Tables

**Table 1:** Information on samples as stated on the product packaging at time of purchase.

Name	Code	Energy (KJ)	Protein (g)	Fat (g)	Carbohydrates (g)	Fiber (g)	Sodium (g)	Calcium (mg)
Daily Box Organic Oat Milk	M1	234	1.5	2	7.1	1.7	41	/
Daily Box High-Fiber Original Oat Milk	M2	257	1.4	2.8	6.2	3	42	/
Daily Box Golden Oat Milk	M3	222	1.4	2.4	5.9	1.1	25	/
OATLY Standard Oat Milk	M4	270	1	3.8	7.9	1	42	120
OATLY Original Oat Milk	M5	232	0.8	1.5	9.3	0.6	50	120
OATLY Barista Edition Oat Drink	M6	245	1	3	6.5	0.8	46	120
Feichangmai Organic Oat Milk	M7	238	0.9	2.3	8.1	0.1	64	/
Songyuru Low-Fat High-Calcium Oat Milk	M8	176	1	1.4	6	0.7	25	120
Fixbody Oat Milk	M9	279	1.4	3.6	7.2	/	42	/
MILK LAB Oat Milk	M10	246	0.6	2.8	8	<0.5	55	120

**Table 2:** Basic physicochemical properties of samples.

Sample	Brix (%)	TTA (g Lactic Acid/100 mL)	pH	L*	a*	b*
M1	9.63 ± 0.26 ^c^	35.56 ± 3.47 ^ab^	6.99 ± 0.07 ^b^	63.20 ± 0.03 ^b^	2.14 ± 0.22 ^cd^	8.75 ± 0.49 ^c^
M2	9.60 ± 0.91 ^c^	22.85 ± 4.31 ^bc^	6.90 ± 0.09 ^b^	67.52 ± 0.94 ^a^	2.52 ± 0.13 ^cd^	9.09 ± 0.05 ^c^
M3	12.93 ± 0.15 ^ab^	34.81 ± 1.31 ^ab^	6.77 ± 0.01 ^c^	64.14 ± 1.63 ^a^	2.67 ± 0.07 ^c^	9.51 ± 0.11 ^c^
M4	12.20 ± 0.4 ^abc^	28.00 ± 1.31 ^abc^	7.33 ± 0.00 ^a^	67.63 ± 1.64 ^a^	1.52 ± 0.26 ^f^	10.02 ± 0.47 ^c^
M5	9.93 ± 1.07 ^c^	31.02 ± 4.73 ^abc^	6.95 ± 0.02 ^b^	65.08 ± 0.30 ^a^	4.77 ± 0.06 ^a^	13.69 ± 0.18 ^a^
M6	11.77 ± 0.09 ^bc^	40.10 ± 4.73 ^a^	7.26 ± 0.04 ^a^	68.96 ± 0.69 ^a^	2.05 ± 0.24 ^de^	10.38 ± 0.78 ^c^
M7	11.13 ± 0.12 ^bc^	28.75 ± 4.73 ^abc^	6.58 ± 0.01 ^d^	66.86 ± 1.04 ^a^	2.40 ± 0.30 ^cd^	9.47 ± 0.21 ^c^
M8	9.83 ± 0.09 ^c^	33.29 ± 2.62 ^ab^	6.77 ± 0.01 ^c^	59.84 ± 0.31 ^c^	1.60 ± 0.31 ^ef^	8.99 ± 0.30 ^c^
M9	14.77 ± 0.77 ^a^	24.97 ± 9.89 ^bc^	7.00 ± 0.01 ^b^	61.34 ± 1.26 ^b^	4.11 ± 0.09 ^b^	12.93 ± 0.41 ^b^
M10	11.60 ± 2.14 ^bc^	18.92 ± 5.71 ^bc^	6.91 ± 0.01 ^b^	69.36 ± 0.31 ^a^	2.26 ± 0.22 ^cd^	11.68 ± 0.62 ^c^

Note: Values (mean ± SD, *n* = 3) in the same line followed by a different letter are significantly different (*p* < 0.05, post hoc Tukey test). Commercial oat milk products: M1, Daily Box Organic; M2, Daily Box High-Fiber Original; M3, Daily Box Golden; M4, OATLY Standard; M5, OATLY Original; M6, OATLY Barista Edition; M7, Feichangmai Organic; M8, Songyuru Low-Fat High-Calcium; M9, Fixbody; M10, MILK LAB.

**Table 3:** Apparent viscosity and particle size distribution.

Sample	Apparent Viscosity					Particle Size		
	*n*	*K* (Pa·S^n^)	η_50_ (mPa·s)	η_100_ (mPa·s)	R^2^	*D_[3,2]_* (μm)	*D_[4,3]_* (μm)	Span
M1	0.37 ± 0.02 ^a^	0.36 ± 0.05 ^a^	30.46 ± 2.64 ^a^	19.65 ± 1.64 ^a^	0.997	12.56 ± 0.27 ^a^	18.44 ± 0.72 ^b^	1.42 ± 0.06 ^g^
M2	0.33 ± 0.01 ^a^	0.30 ± 0.04 ^a^	21.68 ± 2.02 ^c^	13.62 ± 1.18 ^c^	0.999	11.27 ± 0.47 ^b^	15.59 ± 0.79 ^c^	1.38 ± 0.01 ^h^
M3	0.34 ± 0.02 ^a^	0.26 ± 0.00 ^a^	19.81 ± 1.39 ^c^	12.53 ± 1.06 ^c^	0.998	11.04 ± 0.33 ^c^	14.61 ± 0.47 ^d^	1.31 ± 0.00 ^i^
M4	0.38 ± 0.17 ^a^	0.09 ± 0.06 ^b^	6.34 ± 0.32 ^b^	4.13 ± 0.44 ^b^	0.946	2.51 ± 0.03 ^f^	3.00 ± 0.04 ^g^	1.21 ± 0.01 ^j^
M5	0.34 ± 0.04 ^a^	0.07 ± 0.02 ^b^	5.26 ± 0.85 ^b^	3.33 ± 0.47 ^b^	0.945	0.45 ± 0.01 ^g^	4.60 ± 0.09 ^f^	15.93 ± 0.34 ^a^
M6	0.18 ± 0.07 ^a^	0.15 ± 0.06 ^b^	5.86 ± 0.88 ^b^	3.31 ± 0.38 ^b^	0.986	0.42 ± 0.01 ^h^	2.01 ± 0.01 ^h^	6.04 ± 0.05 ^b^
M7	0.53 ± 0.01 ^a^	0.05 ± 0.00 ^b^	7.87 ± 0.37 ^b^	5.68 ± 0.22 ^b^	0.907	3.64 ± 0.02 ^e^	6.13 ± 0.15 ^e^	2.20 ± 0.07 ^f^
M8	0.29 ± 0.12 ^a^	0.05 ± 0.03 ^b^	2.63 ± 0.28 ^b^	1.61 ± 0.06 ^b^	0.999	0.14 ± 0.00 ^j^	1.34 ± 0.01 ^i^	5.36 ± 0.06 ^c^
M9	0.35 ± 0.04 ^a^	0.30 ± 0.12 ^a^	22.64 ± 6.68 ^c^	14.33 ± 3.96 ^c^	0.996	6.00 ± 0.23 ^d^	35.87 ± 0.84 ^a^	2.69 ± 0.02 ^e^
M10	0.29 ± 0.24 ^a^	0.07 ± 0.05 ^b^	3.20 ± 0.60 ^b^	1.94 ± 0.18 ^b^	0.994	0.28 ± 0.00 ^i^	0.90 ± 0.03 ^j^	4.86 ± 0.13 ^d^

Note: *n* = Flow behavior Index; *K* = Consistency index; η_50_ = Apparent viscosity of 50 s; η_100_ = Apparent viscosity of 100 s; R^2^ = Coefficient of determination; Values (mean ± SD, n = 3) in the same line followed by a different letter are significantly different (*p* < 0.05, post hoc Tukey test). Commercial oat milk products: M1, Daily Box Organic; M2, Daily Box High-Fiber Original; M3, Daily Box Golden; M4, OATLY Standard; M5, OATLY Original; M6, OATLY Barista Edition; M7, Feichangmai Organic; M8, Songyuru Low-Fat High-Calcium; M9, Fixbody; M10, MILK LAB.

**Table 4:** Volatile compounds of oat milk samples and identification methods.

No.	CAS	Name	RI	Identification Method	Odor Description
**1**	99-87-6	p-Cymene	1025	MS/RI	solvent, gasoline, citrus
**2**	99-85-4	γ-Terpinene	1060	MS/RI	gasoline, turpentine
**3**	98-86-2	Acetophenone	1066	MS/RI	must, flower, almond
**4**	98-01-1	Furfural	833	MS/RI	bread, almond, sweet
**5**	80-56-8	α-Pinene	937	MS/RI	cedarwood, pine
**6**	7786-61-0	4-Vinylguaiacol	1316	MS/RI	pine, turpentine
**7**	693-54-9	2-Decanone	1193	MS/RI	fat, fruit
**8**	66-25-1	Hexanal	801	MS/RI	apple, fat, fresh, green oil
**9**	5989-27-5	D-Limonene	1031	MS/RI	pine, herbal, peppery
**10**	544-12-7	3-Hexen-1-ol	856	MS/RI	peach, fat
**11**	4861-58-9	2-Pentyl-thiophene	1170	MS/RI	sweet, fruit
**12**	4312-99-6	1-Octen-3-one	979	MS/RI	mushroom, metal
**13**	38284-27-4	3,5-Octadien-2-one	1091	MS/RI	fruity, fatty, mushroom
**14**	3777-69-3	2-Pentyl-furan	993	MS/RI	green bean, butter
**15**	3658-77-3	Furaneol	1070	MS/RI	caramel
**16**	3391-86-4	1-Octen-3-ol	980	MS/RI	cucumber, fat, floral, mushroom
**17**	2628-17-3	4-Vinylphenol	1223	MS/RI	almond shell
**18**	24295-03-2	2-Acetylthiazole	1021	MS/RI	roast, nut, sulfur
**19**	22047-25-2	Acetylpyrazine	1023	MS/RI	nut, crushed bug
**20**	1669-44-9	3-Octen-2-one	1040	MS/RI	green, nut, rose
**21**	143-08-8	1-Nonanol	1173	MS/RI	fat, floral, green, oil
**22**	124-19-6	Nonanal	1104	MS/RI	floral, green, fat, lemon
**23**	124-13-0	Octanal	1003	MS/RI	fruity, fatty, citrus, honey
**24**	121-33-5	Vanillin	1404	MS/RI	vanilla
**25**	118-71-8	Maltol	1110	MS/RI	caramel
**26**	112-31-2	Decanal	1206	MS/RI	soap, orange peel, tallow
**27**	111-87-5	1-Octanol	1070	MS/RI	bitter almond, fat, floral
**28**	111-71-7	Heptanal	901	MS/RI	citrus, fat, green, nut
**29**	111-70-6	1-Heptanol	970	MS/RI	almond, fat, fruit
**30**	111-27-3	1-Hexanol	868	MS/RI	resin, flower, green
**31**	110-43-0	2-Heptanone	891	MS/RI	fruity, sweet, coconut, woody
**32**	110-38-3	Ethyl decanoate	1396	MS/RI	grape
**33**	108-50-9	2,6-Dimethyl-pyrazine	916	MS/RI	roasted nut, cocoa, roast beef
**34**	106-32-1	Ethyl octanoate	1196	MS/RI	fruit, fat
**35**	104-61-0	Dihydro-5-pentyl-2(3H)-Furanone	1364	MS/RI	coconut, peach
**36**	104-50-7	5-Butyldihydro-2(3H)-furanone	1261	MS/RI	coconut
**37**	100-52-7	Benzaldehyde	962	MS/RI	bitter almond, burnt sugar

Note: Odor description was obtained from the literature. Identification methods of volatile compounds compared with mass spectrum (MS) in Wiley mass database, compared to the Kovats Retention Index (RI) on a DB-wax column with authentic chemical.

## Data Availability

The original contributions presented in the study are included in the article and Supplementary Materials, further inquiries can be directed to the corresponding authors.

## Supplementary Materials

The following supporting information can be downloaded at: https://www.mdpi.com/article/10.3390/foods15050936/s1, Table S1: The performance description of electronic nose sensors. Table S2: Definition of sensory attributes evaluated in oat milk samples. Table S3: Analysis of volatile compounds in oat milk samples (mg/kg).

## Abbreviations

The following abbreviations are used in this manuscript:

ANOVA	One-way Analysis of Variance
CAGR	Compound Annual Growth Rate
GC-MS	Gas Chromatography-Mass Spectrometry
GC-MS-O	Gas Chromatography–Mass Spectrometry–Olfactometry
HSD	Honest Significant Difference
HS-SPME	Headspace Solid-phase Microextraction
JAR	Just-about-right
OAV	Odor-activity Values
PCA	Principal Components Analysis
PBMs	Plant-based milk beverages
RATA	Rate-All-That-Applied
TTA	Total titratable acidity
VOCs	Volatile Organic Compounds
