# Supplementary material for: Multi-Modal Data Fusion for Quality Discrimination and Flavor Analysis of Commercial Oat Milk
Source: Foods. 2026 Mar 7;15(5):936. doi: 10.3390/foods15050936 (PMC12984210; doi:10.3390/foods15050936)
Supplement: Supplementary file 1 [file foods-15-00936-s001.zip › foods-4101089-supplementary.pdf]

Table S1 The performance description of electronic nose sensors.

Sensors	Performance descriptions
W1C	Aromatic components, benzene compounds
W1S	Short chain alkanes such as methane
W1W	Sulfur inorganic compounds
W2S	Alcohols, aldehydes, ketones, ethers
W2W	Aromatic components, sulfur organic compounds
W3C	Aromatic components, ammonia
W3S	Sensitive to long chain alkanes
W5C	Aromatic components, alkanes
W5S	Nitrogen oxides
W6S	Sensitive to hydrogen

Table S2. Definition of sensory attributes evaluated in oat milk samples.

Attribute	Definition
Whiteness	The degree to which the sample appears bright and white in color.
Yellowness	The intensity of yellow coloration perceived in the sample.
Grayness	The extent to which the sample appears dull or grayish.
Oatiness	The characteristic aroma or flavor reminiscent of oats or cereal grains, representing the typical identity of oat-based products.
Burntness	The perception of a roasted or over-heated note resembling burnt sugar, caramel, or scorched cereal.
Beaniness	A legume-like aroma or flavor often associated with soy or pulses.
Sweet-scentedness	The pleasant and mild aroma associated with sweetness, often linked to vanilla, floral, or caramel-like notes.
Sweetness	The basic taste sensation elicited by sugars or sweet-tasting compounds.
Bitterness	The basic taste sensation perceived as sharp or unpleasant.
Richness	The perception of flavor fullness and intensity.
Thickness	The perceived viscosity or body of the sample in the mouth.
Graininess	The tactile sensation of fine particles or residues on the tongue.

Table S3. Analysis of volatile compounds in oat milk samples (ug/kg).

Compound	M1	M2	M3	M4	M5	M6	M7	M8	M9	M10
p-Cymene	ND	ND	ND	ND	ND	2.55±0.94^{b}	10.18±1.45^{a}	ND	0.32±0.01^{b}	ND
γ-Terpinene	1.90±0.11^{b}	2.69±0.78^{ab}	ND	ND	3.20±0.20^{a}	0.40±0.13^{c}	0.67±0.13^{c}	ND	ND	ND
Acetophenone	ND	0.15±0.05^{c}	0.34±0.06^{b}	0.15±0.17^{b}	ND	ND	ND	ND	ND	1.10±0.04^{a}
Furfural	13.12±2.89^{b}	ND	13.03±1.52^{b}	ND	ND	ND	1.52±0.46^{b}	ND	115.39±5.61^{a}	ND
α-Pinene	ND	ND	ND	ND	49.89±2.80^{a}	32.85±3.66^{b}	ND	ND	ND	ND
4-Vinylguaiacol	ND	ND	ND	1.00±1.10^{b}	1.99±0.23^{a}	ND	0.96±0.02^{b}	ND	0.30±0.03^{c}	ND
2-Decanone	0.23±0.09^{c}	ND	0.64±0.16^{b}	0.25±0.28^{c}	ND	0.29±0.04^{c}	0.69±0.15^{b}	ND	1.63±0.39^{b}	2.14±0.10^{a}
Hexanal	11.02±0.76^{b}	11.23±2.37^{b}	21.46±1.54^{a}	18.66±1.98^{a}	ND	5.91±1.90^{c}	ND	ND	ND	9.49±0.34^{b}
D-Limonene	28.50±1.35^{b}	85.86±2.11^{a}	11.65±1.70^{c}	8.94±0.78^{c}	4.88±0.14^{d}	ND	1.43±0.31^{e}	2.53±0.49^{de}	1.81±0.90^{de}	ND
3-Hexen-1-ol	ND	ND	ND	ND	5.03±0.73^{a}	ND	ND	0.58±0.08^{b}	ND	ND
2-Pentyl-thiophene	0.69±0.08^{ab}	0.36±0.02^{ab}	0.86±0.18^{ab}	1.1.69±0.26^{b}	0.18±0.65^{c}	0.14±0.01^{c}	0.46±0.21^{ab}	0.27±0.07^{c}	2.49±0.73^{a}	0.59±0.03^{ab}
1-Octen-3-one	ND	ND	ND	ND	ND	1.27±0.44	ND	ND	ND	ND
3,5-Octadien-2-one	3.16±0.51^{d}	8.41±1.00^{bc}	12.98±1.77^{a}	9.98±1.06^{b}	1.53±0.04^{d}	ND	8.33±1.10^{bc}	2.64±0.16^{d}	14.12±0.87^{a}	7.32±0.83^{c}
2-Pentyl-furan	18.47±0.89^{e}	32.90±1.26^{d}	168.15±4.15^{a}	14.19±1.18^{g}	9.22±1.51^{h}	19.21±1.52^{e}	16.65±1.29^{ef}	20.39±0.62^{e}	48.30±0.74^{b}	39.99±1.17^{c}
Furaneol	ND	ND	ND	ND	ND	ND	ND	ND	10.00±0.99	ND
1-Octen-3-ol	3.24±0.31^{b}	3.10±0.68^{bc}	9.43±1.24^{a}	9.00±0.65^{a}	1.42±0.08^{cd}	0.35±0.06^{d}	ND	0.33±0.06^{d}	4.45±0.59^{b}	ND
4-Vinylphenol	ND	ND	ND	ND	6.78±0.61	ND	ND	ND	ND	ND
2-Acetylthiazole	ND	ND	ND	ND	29.23±0.77^{a}	ND	5.19±0.45^{b}	ND	ND	ND
Acetyl pyrazine	ND	ND	ND	ND	54.24±0.28	ND	ND	ND	ND	ND

Table S3 (Continued)

Compound	M1	M2	M3	M4	M5	M6	M7	M8	M9	M10
3-Octen-2-one	2.03±0.79[ab]	1.62±0.64[b]	3.38±0.41[a]	3.25±0.69[a]	ND	ND	1.77±0.46[b]	ND	1.68±0.41[b]	ND
1-Nonanol	6.43±1.40[c]	2.82±0.16[d]	9.05±0.92[b]	ND	ND	ND	9.38±1.17[b]	ND	ND	14.70±0.70[a]
Nonanal	9.30±0.39[c]	12.13±0.37[c]	20.36±0.32[b]	10.44±0.78[c]	2.71±0.50[e]	4.78±0.67[de]	ND	8.61±0.33[cd]	32.58±2.31[a]	18.30±3.33[b]
Octanal	3.05±0.51[c]	1.59±0.20[de]	2.62±0.30[cd]	2.33±0.25[cd]	ND	9.74±0.88[a]	ND	1.36±0.10[f]	4.42±0.22[b]	4.60±0.44[b]
Vanillin	ND	ND	ND	ND	1.58±0.26[b]	ND	ND	ND	4.50±0.31[a]	ND
Maltol	ND	ND	ND	ND	59.47±3.16	ND	ND	ND	ND	ND
Decanal	0.37±0.66[b]	ND	2.20±0.30[a]	ND	ND	ND	ND	ND	ND	ND
1-Octanol	ND	ND	0.67±0.04[a]	0.45±0.01[b]	ND	ND	ND	0.60±0.13[ab]	ND	ND
Heptanal	5.90±0.46[d]	6.57±0.72[d]	11.52±1.08[b]	3.07±0.74[e]	ND	ND	14.89±0.37[a]	2.37±0.41[e]	9.18±0.66[c]	ND
1-Heptanol	ND	1.10±0.16[d]	2.99±0.67[c]	2.58±0.52[c]	ND	ND	2.96±0.05[c]	ND	9.17±0.66[a]	5.12±0.71[b]
1-Hexanol	3.51±0.52[c]	3.60±0.80[c]	5.26±0.34[b]	5.64±0.19[b]	ND	ND	10.69±0.29[a]	1.44±0.12[d]	3.90±0.06[c]	ND
2-Heptanone	3.34±0.22[c]	5.70±0.34[b]	9.74±0.63[a]	ND	3.32±0.42[c]	ND	6.75±0.43[b]	ND	ND	ND
Ethyl decanoate	1.39±0.20[cd]	ND	2.22±0.11[bc]	ND	ND	1.00±0.05[d]	6.75±0.43[a]	ND	3.00±0.51[b]	ND
2,6-Dimethyl-pyrazine	ND	ND	ND	ND	4.52±0.36[a]	ND	0.27±0.07[c]	1.51±0.06[b]	ND	ND
Ethyl octanoate	ND	ND	ND	ND	12.25±1.06	ND	ND	ND	ND	ND
Dihydro-5-pentyl-2(3H)-Furanone	ND	ND	ND	ND	45.57±1.21[a]	ND	ND	ND	0.84±0.03[b]	ND
5-Butyldihydro-2(3H)-furanone	ND	ND	ND	ND	41.42±0.93	ND	ND	ND	ND	ND
Benzaldehyde	ND	6.08±0.94[d]	6.39±0.30[d]	8.98±0.62[c]	ND	1.84±0.71[e]	ND	4.09±0.18[de]	13.80±0.41[b]	28.57±1.87[a]

Note: ND, not detectable. Different letters on the same raw means that the data are significantly different ($p < 0.05$). NS, not significant effects ($p > 0.05$).
